# Chaotic Image Encryption Algorithm Based on Bit Permutation and Dynamic DNA Encoding

**DOI:** 10.1155/2017/6919675

**Published:** 2017-08-22

**Authors:** Xuncai Zhang, Feng Han, Ying Niu

**Affiliations:** School of Electrics and Information Engineering, Zhengzhou University of Light Industry, Zhengzhou 450002, China

## Abstract

With the help of the fact that chaos is sensitive to initial conditions and pseudorandomness, combined with the spatial configurations in the DNA molecule's inherent and unique information processing ability, a novel image encryption algorithm based on bit permutation and dynamic DNA encoding is proposed here. The algorithm first uses Keccak to calculate the hash value for a given DNA sequence as the initial value of a chaotic map; second, it uses a chaotic sequence to scramble the image pixel locations, and the butterfly network is used to implement the bit permutation. Then, the image is coded into a DNA matrix dynamic, and an algebraic operation is performed with the DNA sequence to realize the substitution of the pixels, which further improves the security of the encryption. Finally, the confusion and diffusion properties of the algorithm are further enhanced by the operation of the DNA sequence and the ciphertext feedback. The results of the experiment and security analysis show that the algorithm not only has a large key space and strong sensitivity to the key but can also effectively resist attack operations such as statistical analysis and exhaustive analysis.

## 1. Introduction

With the rapid development of multimedia technology and network technology, digital image processing has been widely applied to all aspects of human life, such as remote sensing, industrial inspection, medical field, meteorology, communications, reconnaissance, and intelligent robots. As a result, increasing attention has been paid to image information. Additionally, it is more important to protect the security of image data, especially in military, commercial, and medical fields. Image encryption technology has become an effective way to protect the transmission of digital images [1]. Image data has the characteristics of large amounts of data, strong correlations, and high redundancy. The existing classical encryption methods cannot meet the needs of image encryption because of its low efficiency and security.

As a type of complex nonlinear system, chaotic systems have initial value sensitivity, pseudorandomness, and nonperiodicity, which are consistent with the characteristics required for cryptography. A chaotic sequence can be used as a random key, which can achieve the same encryption effect as the first time, and it is not capable of being broken, in theory. Thus, chaotic encryption technology has been widely used in the field of information security, especially in the field of image encryption [2, 3].

At present, most of the confusion and diffusion structure of image encryption algorithms is based on chaotic systems for the use of chaotic sequences, and it is restricted by the computer word length, which can cause degradation in the chaotic dynamics, especially for a low-dimensional chaotic system [4]. This limitation seriously affects the security of the chaotic encryption. Therefore, many scholars use hyperchaos systems to ensure the complexity of the chaotic sequence, to improve the security of the algorithm. However, there is no denying that an encryption algorithm that is composed of a single chaotic map cannot guarantee the security of the encrypted image [5].

DNA is an important carrier of the biological genetic information that is stored in the body, and genetic metabolism plays an important role in the organism. It has a very large scale of parallelism, ultrahigh storage density, and low energy consumption as well as a unique molecular structure and molecular recognition mechanism, which determines its outstanding information storage and information processing ability [6]. DNA has great potential in the field of information security, information hiding, and authentication, which provides a new way for the development of modern cryptography [7–9]. Boneh et al. cracked 56 keys in four months in 1995, which is the first time that DNA was used to crack the traditional encryption standard DES [10]. Subsequently, the development of DNA cryptography research has become a hot topic. In 1999, Gehani and others used DNA as an information carrier, using biochemical technology in the DNA molecule and achieved one of the traditional encryption algorithms [11]. In 2013, Le Goff et al. achieved a 3D-particle array encryption model, and they combined DNA particle technology and thermal shrinkage sheets with DNA polymers fixed on a polyethylene heat-shrinkable film; in this way, they successfully formed a three-dimensional DNA hydrogel particle array size within 100 *μ*m [12]. These DNA encryption algorithms are used to encrypt text information, and it is quite difficult for image information to be directly encrypted.

In recent years, combined with the dual advantage of the DNA molecule and chaotic systems, an image encryption algorithm based on DNA molecules and chaotic systems is presented. In 2012, Liu et al. proposed an image encryption algorithm based on DNA encoding and chaotic map [13]. In 2014, [14] Liu et al. proposed a RGB image encryption algorithm based on DNA encoding and chaos map. In 2015, Wang et al. presented an image encryption technique based on 2D logistic mapping and DNA operations [15]. In 2017, Chai et al. presented an image encryption algorithm that is based on chaos combined with DNA operations [16]. In the same year, we proposed a type of digital image encryption technology based on hyperchaos mapping and DNA sequence library arithmetic to realize a scrambling position transformation of image pixels and the spread of the pixel values [17]. These algorithms displace only the positions of the image pixels and change the gray value. However, the bit's position changes are smaller, and it is not able to achieve the purpose of true diffusion [18]. In the replacement phase, the advantage of the bit replacement is obviously better than pixel permutation, because it not only changes the position but also changes the sizes of the pixels [19].

Therefore, in this paper, a new image encryption algorithm based on chaotic systems and dynamic DNA encoding is proposed. The algorithm uses Keccak to compute the hash value of the given DNA sequence as the initial value of the chaotic map, generating a chaotic index of the image position that performs scrambling, which is coupled with a butterfly network to achieve a level of scrambling. Finally, through the study of the dynamic DNA encoding of images and the operations of a given DNA sequence, the additional use of ciphertext feedback can help to achieve the replacement and diffusion of the pixels, which has further improved the security of the encryption.

## 2. Fundamental Theory

### 2.1. Hyperchaos System

As a type of special nonlinear phenomenon, chaos has good pseudorandomness and unpredictability of the orbit and has extreme sensitivity to initial conditions and structure parameters; in addition, it is iterative and not repetitive and has a series of excellent features, which are widely used toward the secrecy of communication. Compared with a low-dimensional chaotic system, high-dimensional chaotic systems have a more positive Lyapunov exponent and are more complex, and it is more difficult to predict the dynamic characteristics, which can effectively solve the degradation problem of the low-dimensional chaotic system with dynamics characteristics. It also has strong confidentiality, a simple algorithm, and large key space characteristics. In 2005, Lee and others constructed a hyperchaos Chen system via state feedback control, and its equation is

(1) x˙=u1y−x+ω,y˙=u4x−xz+u3y,z˙=xy−u2z,ω˙=yz+rω,

where *x*, *y*, *z*, and *w* are the system state variables and *u*_1_, *u*_2_, *u*_3_, *u*_4_, and *r* are the control parameters of the system. When *u*_1_ = 35, *u*_2_ = 3, *u*_3_ = 12, *u*_4_ = 7, and 0.085 ≤ *r* ≤ 0.798, the system's performance is hyperchaos.

### 2.2. Keccak Algorithm

Keccak is a standard one-way hash function algorithm. The hash functions are designed to take a string of any length as input and produce a fixed-length hash value. When the hash value is attached to the message or stored with the message, the message can be prevented from being modified in the process of storage for transmission. Messages are different; the resulting hash value is also different, and even if there is only one bit of change in the message, the hash value will be completely useless. By using this feature, we can change the pixel value of the image by selecting the appropriate message and using the hash value generated by the Keccak hash function and the operation of the image. At the same time, the hash value is modified to set the initial value and system parameters of the chaotic system, to further improve the security of the encryption. Keccak has no length limit on the upper limit of the input data length, and it can generate arbitrary hash values.

### 2.3. DNA Encoding Algebraic Operations

The DNA molecule is composed of four DNA nucleotides, which are adenine (A), cytosine (C), guanine (G), and thymine (T). For two single-stranded DNA molecules, a stable DNA molecule can be formed by hydrogen bonds between nucleotides. The chemical structure of the base determines the principle of complementary base pairing, and it is also known as the Watson-Crick base pairing principle. In other words, A and T are paired by two hydrogen bonds, and G and C are paired by three hydrogen bonds. The natural combination is quaternary, similar to the binary semiconductor formed by on and off [20]. Therefore, the information can be stored and calculated by using the permutations and combinations of bases.

*(1) DNA Encoding Rule*. If we act according to the encoding rules, A→00, C→01, G→10, T→11, then the complementary number matching is 01*↔*10 and 00*↔*11, and the complementary base pair matching is A*↔*T and C*↔*G. In this case, there are eight encoding combinations that satisfy the complementary pairing rules, as shown in Table 1.

For a gray image, the gray value of each pixel can be represented by an 8-bit binary number. If we use the DNA encoding, then each pixel needs four base sequence encodings. By converting the image matrix to a sequence of DNA, the operators for the sequence of the DNA can be applied to the image processing. To reach the goal of pixel value disturbance, the following base operations and transformation rules are defined at the same time.

*(2) Base Operation Rules*. According to the complementary pairing rules, by encoding A→00, C→01, G→10, and T→11, we can give some base operation rules (see Tables 2–4), according to the different encoding rules, and we can also establish similar operation rules.

## 3. Encryption Algorithms

The digital image encryption is realized by using the hyperchaos Chen system, the Keccak algorithm, bit permutation, a dynamic DNA encoding technique, and its pixel gray value transformation and operation to achieve the purpose of confusion and diffusion, to realize the digital image encryption.

### 3.1. Key Sequence Generation

The nucleic acid database is a database of all known nucleic acid information sets. It contains nucleotide sequences, single nucleotide polymorphisms, structure, properties, and related descriptions. The ID number of a sequence in a database is called the sequence code, which is unique and permanent. With the rapid development of sequencing technology, the size of the nucleic acid database is growing exponentially; thus far, public access to DNA sequences includes more than 163 million sequences. This enormous database is equivalent to a natural password. It provides a new idea and solution for image encryption.

The DNA sequence is mainly used for ciphertext diffusion as well as the generation of hash values. Using the Keccak algorithm to generate the hash value *K* of a DNA sequence, the length of *K* is 512 bits. For example, the hash value of the DNA sequence that is numbered NZ_LOZQ01000068 in the GenBank database is 9caa44db566cfe1f6a98c4991fffe891bb7d7fdf840449a026e923e9feab60b8b7ed7a3933a757358c2c9441366976fab4bda222f9b5e4df814322e0dc12c13f, and it can be expressed as *K* = {*k*_1_, *k*_2_, *k*_3_,…, *k*_512_}, which is divided into 64 groups, and each contains a total of 8 bits, *K* = *k*_1_′, *k*_2_′, *k*_3_′,…, *k*_64_′.

### 3.2. Bit Permutations

Scrambling is an important means to hide plaintext information with an encryption algorithm. The diffusion of text can be achieved through position displacement. The bits permutation provides the functionality of the confusion and diffusion that byte operations cannot achieve.

**Figure 1:**
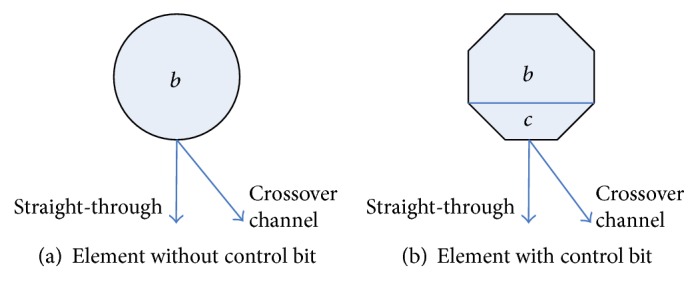
Bit replacement elements.

**Figure 2:**
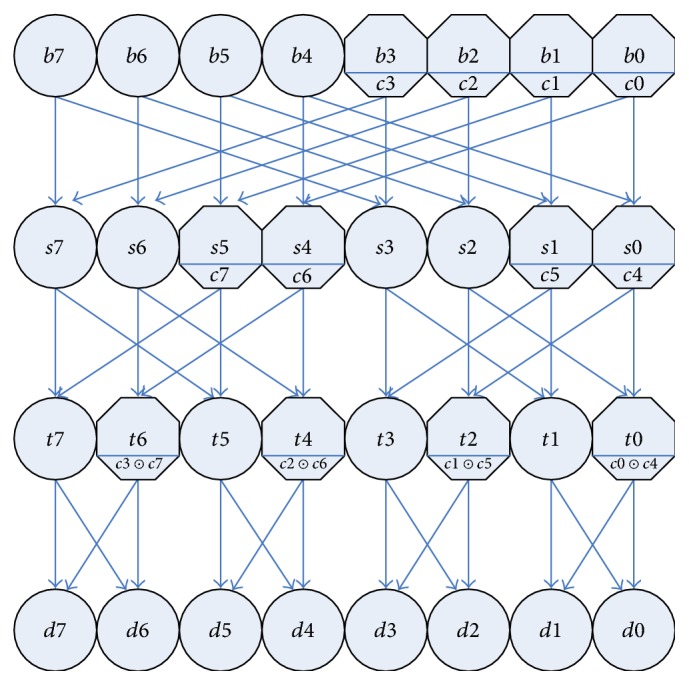
Bit permutation network.

Butterfly is a common network of communication exchanges [21]. The butterfly network and the inverse butterfly network connection can be combined into a type of nonblocking network, which can be implemented from the input to the output side. Therefore, the butterfly permutation network can be used to construct the bit cell to realize any permutation operation. In this paper, two types of switching elements are defined to control bit permutation.  Figure 1 shows, respectively, the round and octagonal elements each of which has straight-through and crossover channels. Each component center contains a bit *b* for replacement. The base of the octagonal element contains a control bit *c*. For the octagonal switching element, when *c* = 1, choose the crossover, and when *c* = 0, choose the straight-through. For the circular element, it is a passive choice of channels, depending on the corresponding channel that is not occupied by the next layer, and it can only choose not to be an occupied channel. According to the design principle of the butterfly network, when calculating from right to left, it always has octagonal elements to make the choice of channel first. Once the octagonal element operation is finished, the remaining round elements have only one channel to be selected. Once the octagonal element operation is completed, the remaining round elements have only one channel to be selected. For example, given the replacement byte *B* = 10100101 and the control byte *C* = 10011001, the middle byte *S* = 00111100, byte *T* = 10011001, and byte *D* = 01100110, to achieve the bit replacement from byte 10100101 to 01100110. To achieve the purpose of replacement, this paper implements three-level scrambling (as shown in Figure 2), and through the improvement of the butterfly network, we need only a control word to control the bit replacement of a given byte.

After the image has been dislocated, the pixels have changed, and the pixel values have been changed to further enhance the security.

### 3.3. Pixel Position Scrambling

*(1) The Generation of Chaotic Sequences*. Based on the preceding hash value *K* = *k*_1_′, *k*_2_′, *k*_3_′,…, *k*_64_′, the initial values of the chaotic system are computed by the following formulas for *x*_0_, *y*_0_, *z*_0_, and *w*_0_:

(2) hi=kj+1′⊕kj+2′⊕kj+3′⊕kj+4′⊕kj+5′⊕kj+6′256,x0=x0′+absroundh1−h1,y0=y0′+absroundh2−h2,z0=z0′+absroundh3−h3,w0=w0′+absroundh4−h4,

where *j* = 6(*i* − 1), *i* = 1,2, 3,4; *x*_0_′, *y*_0_′, *z*_0_′, *w*_0_′ are the initial given values.

The given initial state values for *x*_0_, *y*_0_, *z*_0_, and *ω*_0_ are chosen such that the chaotic system is in a hyperchaotic state, and through iterations we can generate 4 given length chaotic sequences; we remove the start-end data from the sequence and take out *L* unrepeatable values. Then, we can obtain four discrete real numeric hyperchaos sequences, *A*_1_ : {*a*_11_, *a*_12_,…, *a*_1*L*_}; *A*_2_ : {*a*_21_, *a*_22_,…, *a*_2*L*_}; *A*_3_ : {*a*_31_, *a*_32_,…, *a*_3*L*_}; and *A*_4_ : {*a*_41_, *a*_42_,…, *a*_4*L*_}. To unify the value range of the real sequence, only by obtaining the decimal part of the 4 sequences can we obtain the new sequences, which are *B*_1_ : {*b*_11_, *b*_12_,…, *b*_1*L*_}; *B*_2_ : {*b*_21_, *b*_22_,…, *b*_2*L*_}; *B*_3_ : {*b*_31_, *b*_32_,…, *b*_3*L*_}; and *B*_4_ : {*b*_41_, *b*_42_,…, *b*_4*L*_}. Then, we have

(3) B1=A1−A1,B2=A2−A2,B3=A3−A3,B4=A4−A4.

Here, [*x*] represents the integer part of *x*.

*(2) Global Scrambling of the Pixel Positions*. The image scrambling technology is to improve the robustness of the hidden carrier by a rearrangement of the image pixel matrix and to destroy the correlation of the image matrix. Thus, image scrambling is a very common technique in information hiding. This approach will enable the encryption of the information to achieve the purpose of safe transmission of images.

We are given a two-dimensional matrix *P* and given that image scrambling is to find a two-dimensional reversible mapping *T* (scrambling matrix). When the matrix *P* is transformed by *T*, then we can obtain a two-dimensional matrix *P*′. The correspondence between *P* and *P*′ is

(4) $$\begin{array}{c} p_{i,j}=p_{u,v}^{\prime }, \end{array}$$

where *u* = div(*T*_*i*,*j*_ − 1, *M*) + 1, *v* = mod(*T*_*i*,*j*_ − 1, *M*) + 1; *i* ∈ {1,2,…, *M*}, *j* ∈ {1,2,…, *N*}. *M* and *N* are the number of rows and columns of the two-dimensional matrix *P*.

According to the sequence *B*_1_ produced by the Chen system, the permutation index sequence *X* is obtained in ascending order, and *X* is populated according to the *M* value of each line to obtain the permutation matrix *T*, which is used for the position scrambling of the image pixels. The corresponding relation between *T*_*i*,*j*_ of each element in *T* and *T*_*k*_ of each element in *X* is as follows:

(5) $$\begin{array}{c} T_{i,j}=X_{\left(i-1\right)*j+j}, \end{array}$$

where *i* ∈ {1,2,…, *M*}, *j* ∈ {1,2,…, *N*}.

*(3) Permutation and Diffusion of Pixels in the Subimages*. The operation of the pixel positions in the global scrambling stage will achieve global scrambling of the image pixel position, in such a way as to break the correlation of the adjacent pixels. We only scramble the location of the image, without changing the pixel value, which would not be safe because it would make it difficult to resist a plaintext attack. To enhance the security of the encryption algorithm by combining with the principle of visual cryptography, the image is divided into two subimages, and the pixel value is further scrambled to achieve pixel diffusion.

For the grayscale image *P*, each pixel value can be represented by an 8-bit binary sequence, namely, *P*_*i*,*j*_ = (*b*_8_*b*_7_*b*_6_*b*_5_*b*_4_*b*_3_*b*_2_*b*_1_)_*i*,*j*_, where *b*_*k*_ ∈ {0,1}, *k* = 1,2,…, 8. Here, *b*_8_ represents the highest bit, and *b*_1_ represents the lowest bit. The 1, 3, 5, and 7 bits in the binary numbers of each pixel are taken out as the low 4 bits of a byte, with a high 4-bit complement 0; then, it forms a byte and rebuilds the submatrices with new pixel values in the corresponding positions. Then, we can obtain the matrix Sub_I. Similarly, the 2, 4, 6, and 8 bits in the binary number of each pixel are taken out as the low 4 bits of a byte, with a high 4-bit complement 0; then, it forms a byte and rebuilds the submatrices as new pixel values in the corresponding positions. Then, we can obtain the matrix Sub_II. Such a grayscale image can be determined by the submatrices in these 2 subgraphs, and the information for each pixel value in the image is distributed among the two submatrices.

According to the sequence *B*_2_ generated by the Chen system, the sequences *y*_1_ and *y*_2_, whose lengths are *M* and *N*, have been intercepted successively from the sequence *B*_2_. Then, the two sequences are sorted in ascending order, and we obtain two ordered sequences, *y*_1_′ and *y*_2_′. We find the positions of the values of the sequences *y*_1_′ and *y*_2_′ in the sequences *y*_1_ and *y*_2_, respectively, and mark down the transformed positions *YM* and *YN*. First, all of the rows of the submatrix Sub_I perform a left cyclic shift; for example, all of the elements of the *i*th row perform a left cyclic shift *YM*_*i*_ times. Then, all of the columns of the submatrix Sub_I are moved circularly upward; for example, all of the elements of the *j*th column are moved circularly upward *YN*_*j*_ times. Similarly, according to the chaos-generated sequence *B*_3_, the sequences *y*_3_ and *y*_4_, whose lengths are *M* and *N*, have been intercepted successively from the sequence *B*_3_. Then, the two sequences are sorted in ascending order, and we obtain two ordered sequences, *y*_3_′ and *y*_4_′. We find the positions of the values of the sequences *y*_3_′ and *y*_4_′ in the sequences *y*_3_ and *y*_4_, respectively, and mark down the transformed positions *ZM* and *ZN*. According to the index sequences *ZM* and *ZN*, the Sub_II of the matrix is a cyclic shift to the right and downward.

After the scrambling of each of the submatrices Sub_I and Sub_II, the two submatrices are restored to an image matrix *I*_4_. The purpose of the ciphertext scrambling and diffusion is realized.

### 3.4. Pixel Substitution and Ciphertext Diffusion

Pixel scrambling quickly disrupts the position of the image through the initial change in the matrix, destroying the correlation between adjacent pixels, but it is unable to effectively resist partial cryptography attacks, and further, through pixel substitution and ciphertext diffusion, it can thoroughly confuse the relationship between the plaintext image and the ciphertext image.

It is possible to achieve a better confusing effect by using complex nonlinear alternative transformations. Alternative encryption means to include modulo arithmetic and addition operations, which can cause the pixel values to be associated with other values and, thus, make the distribution of the pixel values more uniform and eliminate the texture feature of the replacement image.

*(1) Pixel Replacement*. The image is converted into the DNA sequence DNA_S by encoding rules, and then, the algebraic operation is performed with the given DNA sequence SQ, which achieves the purpose of pixel substitution. The DNA sequence operation can be addition, subtraction, or the XOR operation. Here is a dynamic DNA encoding technique for each pixel, and the dynamic DNA encoding technique is based on the position where the pixel of the matrix *I* to be encoded is located and the previously generated hash value. The DNA encoding rule selected for the pixels *I*_*i*,*j*_ is calculated as follows:

(6) Ri,j=modi−1∗N+j,8⊕Bin2decksks+1ks+2,

where *i* ∈ {1,2,…, *M*}, *j* ∈ {1,2,…, *N*}, *s* = mod((*i* − 1)*∗N* + *j* − 1,510) + 1, *k*_*s*_*k*_*s*+1_*k*_*s*+2_ consists of three bits of the hash value *K*: *s* and *s* + 1 and *s* + 2 bits. Bin2dec(*k*_*s*_*k*_*s*+1_*k*_*s*+2_) is a function that converts a three-bit binary number into a decimal number.

Since each pixel value can be represented by an 8-bit binary, each pixel is encoded as 4 bases. Then, the encoded DNA sequence length is 4 × *M* × *N*. For example, the pixel value of the element in the thirty-seventh row and the fifty-fourth column for the original image is 108, which can be expressed in binary [01101100], and according to the dynamic encoding technology, the rule *R*_37,54_ = 8 should be selected; it is encoded by the DNA encoding rule 8, and the DNA sequence of the pixel is [GCAT].

The sequence DNA_S is algebraically manipulated with the given DNA sequence SQ. Algebraic operations can be one of the operations in Tables 2, 3, or 4. For example, select the XOR operation in Table 2:

(7) $$\begin{array}{c} DNA\_SD\left(\;f\;\right)=DNA\_S\left(\;f\;\right)⊕SQ\left(\;f\;\right), \end{array}$$

subject to *f* = 1,2, 3,…, 4 × *M* × *N*.

Finally, we use DNA encoding rule 1 to decode the DNA sequence DNA_SD, and the image matrix is restored.

*(2) Ciphertext Diffusion*. The diffusion operation of the ciphertext can spread the tiny changes of the plaintext into the whole ciphertext, in such a way that the relationship between the plaintext image and ciphertext image can be disturbed. The image matrix is transformed into the one-dimensional sequence SI = {si_1_, si_2_, si_3_,…, si_*M*×*N*_}, with the length of *M* × *N* in the order of row priority, and the one-dimensional sequence after the ciphertext diffusion is SD = {sd_1_, sd_2_, sd_3_,…, sd_*M*×*N*_}. Thus, the ciphertext diffusion formula is as follows:

(8) $$\begin{array}{c} sd_{t+1}={si}_{t}⊕{sd}_{t-1}, \end{array}$$

where the initial element sd(0) = *k*_1_′, *t* = 1,2,…, *M∗N*, and *k*_1_′ is the first 8 bits of the previously generated hash value *K*. After the diffusion, the SD sequence is transformed into a two-dimensional matrix of size *M* × *N*.

### 3.5. Encryption Algorithm

**Figure 3:**
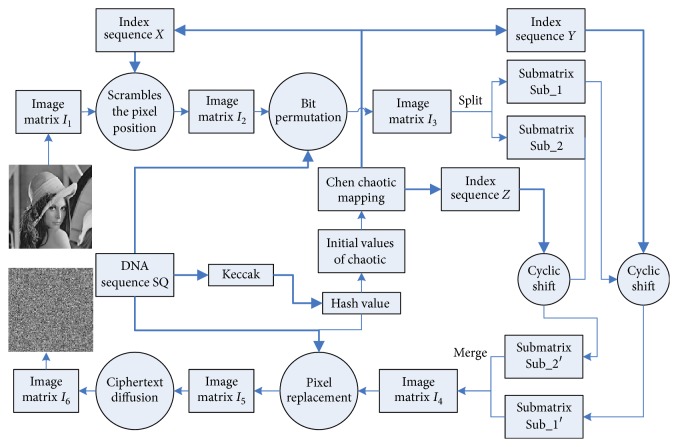
Description of the encryption process.

The digital image encryption algorithm proposed in this paper includes the following: first, bit permutation, the use of the butterfly network to achieve each pixel bit position permutation; second, pixel location scrambling transforms. The image pixel location will be changed through the displacement index created by the hyperchaotic Chen system to constitute the necessary permutation indices. Third, there are pixel substitution and ciphertext diffusion. The value of each pixel of the original image is converted into a DNA sequence, and the sequence of the DNA coding sequence library is calculated; then, it iterates through the ciphertext feedback. The encrypted flowchart is shown in Figure 3. The specific steps are as follows.

*Input*. The input is grayscale image *I* and initial value of the parameter. 

*Output*. The output is encrypted image.

1. Convert the grayscale image *I* into a two-dimensional matrix *I*_1_ with the size *M* × *N*.
2. Download the DNA sequence SQ whose ID number is NZ_LOZQ01000068 from the GenBank database, using the Keccak algorithm to calculate the hash value *K* for the DNA sequence SQ, and generate the chaotic initialization parameters.
3. According to sequence *B*_1_ generated by the Chen system, the permutation index sequence *X* is obtained in ascending order, and the sequence *X* is filled with each row *M*. Thus, the permutation matrix *T* is obtained, and the pixel position in the image matrix *I*_1_ is scrambled with the matrix *T*; then, we can obtain the scrambled matrix *I*_2_.
4. According to the bit permutation principle described in Section 3.2, the improved butterfly network is used to select the DNA encoding rule 1, and the DNA sequence SQ is transformed into a binary number as the control bit. Then, we can achieve bit permutation and obtain the new matrix *I*_3_.
5. According to the subgraph scrambling and diffusion technique described in Section 3.3, the matrix *I*_3_ is divided into two submatrices, and the sequences *B*_2_ and *B*_3_ are generated by the chaotic system. Then, the two submatrices are restored to a matrix after the scrambling and diffusion, respectively, and matrix *I*_4_ is obtained.
6. Using the dynamic DNA encoding technique, the image matrix *I*_4_ is transformed into DNA sequences, and the pixel substitution technique is realized by algebraic operations given the DNA sequence SQ (4 × *M* × *N* base sequence from *S*). Once again, we select an encoding rule to decode the DNA sequence DNA_SD to the image matrix and obtain the matrix *I*_5_.
7. According to the ciphertext diffusion technique described in Section 3.4, the image encryption matrix is *I*_6_, and the output is obtained by the XOR operation with the ciphertext of the previous pixel.

The decryption algorithm is the inverse process of the above process. This process is no longer elaborated.

This algorithm can also be applied to color image encryption, by processing only the values of the pixel RGB decomposition.

## 4. Experimental Results and Safety Analysis

**Figure 4:**
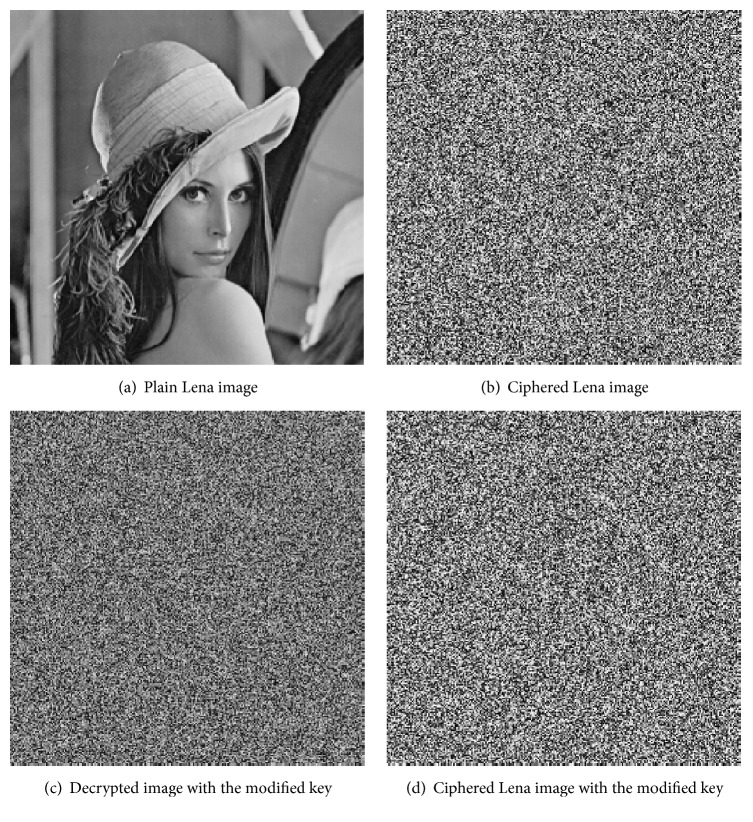
Lena image and ciphered Lena.

Aiming at the algorithm proposed in this paper, the feasibility of the algorithm is verified by MATLAB software programming. This paper adopts a Lena grayscale image with the size 256*∗*256. The original and encrypted images are shown in Figure 4.

### 4.1. Key Space and Its Sensitivity Analysis

The key used in this paper is mainly used for the scrambling and diffusion of the pixels, namely, the following: the Chen system initial parameter *r* = 0.6, the DNA sequence ID number is NZ_LOZQ01000068 in the nucleic acid database, parameters *x*_0_′ = *y*_0_′ = *z*_0_′ = *w*_0_′ = 1, and the starting position is *R* = 1. Other parameters are generated by the hash value of the DNA sequence. The original image and the encrypted image are shown in Figure 4.

If the computation precision is 10^–14^, then the key space can reach 10^100^, which shows that the algorithm has sufficient space to resist an exhaustive attack.

To test the sensitivity of the key, the initial value of the *x*_0_′ is increased by 0.0000000001, and the other keys are unchanged in the Chen system. Using the modified key to decrypt the encrypted image, the decryption results are shown in Figure 4(c). It can be seen that the key to the minor changes cannot correctly decrypt the original image. Furthermore, using the modified key to encrypt the image, the encrypted images shown in Figures 4(d) and 4(b) were compared between the two cipher images that correspond to different pixel rates above 99.5%. The algorithm has strong key sensitivity, and it can resist violent attacks; it has good key security for such attacks.

### 4.2. Gray Histogram Analysis

**Figure 5:**
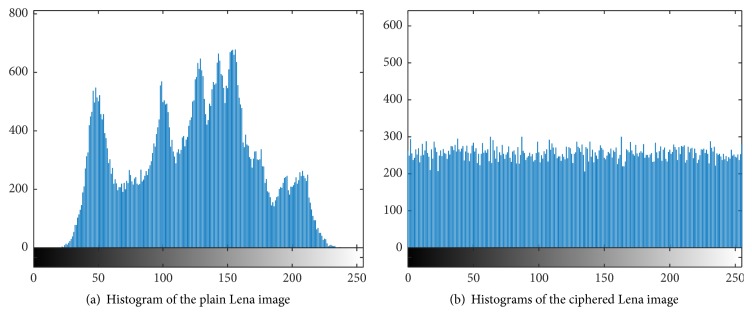
Histogram of the plain Lena image and ciphered Lena image.

The statistical information of the image can reveal the distribution of the gray value of the original image to a certain extent, and whether it can change the statistical distribution of the original image is also an important indicator of the image encryption. The purpose of this algorithm is to strike the attack side against a grayscale statistical attack. As shown in Figure 5, it can be concluded from the experimental results that the XOR processing and the permutation operation make the grayscale distribution of the encrypted image very uniform, which shows that the algorithm has a good ability to resist statistical analysis in such a way that the attacker cannot analyze the original gray value distribution range.

### 4.3. Correlation Coefficient Analysis

**Figure 6:**
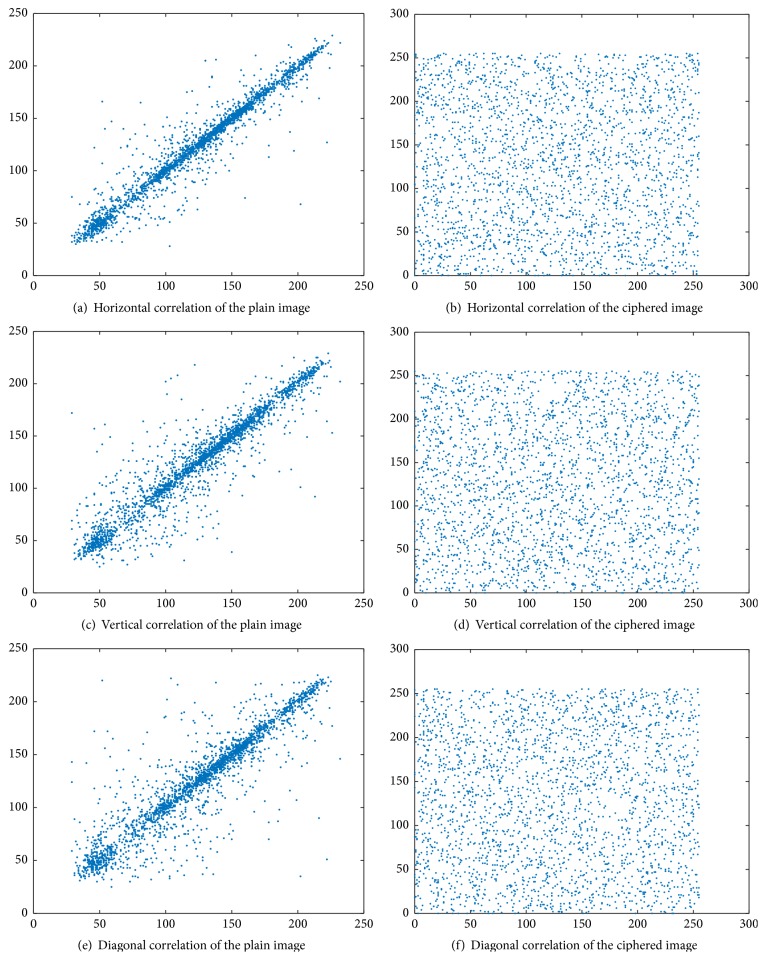
Correlation Analysis of Lena as a ciphered image in three directions.

The correlation between the pixels in the original image is relatively large, and to prevent the statistical analysis, we must reduce the correlation of adjacent pixels. We randomly select from the original image and encrypted image each pixel to 2500-pixel correlation, observing the horizontal and vertical and diagonal directions, as shown in Table 5. As seen from Table 5, there is a significant correlation between the image pixels before the encryption. After the encryption, the correlation between the pixels is greatly reduced. This finding indicates that the adjacent pixels are not related to each other, and the statistical characteristics of the original image have been spread to the random ciphertext image. Table 5 and Figure 6 show the correlation between the original image and the adjacent pixels of the encrypted image.

The performance for ciphered image of Lena is compared with that of Ye's algorithm [22], X. Wang and Q. Wang's algorithm [23], and Liu et al.'s algorithm [13], which are listed in Table 5. It shows that our algorithm can get good encryption effect.

### 4.4. Information Entropy Analysis

Information entropy is a measure of uncertainty. The formula is as follows:

(9) $$\begin{array}{c} H\left(\;m\;\right)=-\overset{2^{N}-1}{\underset{k=0}{\sum }}p\left(\;m_{i}\;\right)\log_{2}\;p\left(\;m_{i}\;\right). \end{array}$$

Here, *p*(*m*_*i*_) represents the probability that the information *m*_*i*_ appears. For grayscale images, the information *m* has 256 states, the minimum value is 0, and the maximum is 255. According to the above equation, when the information entropy is 8, the information is completely random. In other words, the greater the entropy of the ciphertext information is, the more secure the information is. The information entropy of the cryptographic image obtained by encrypting the Lena image is 7.990, which indicates that the information leakage of the ciphertext is very small. The information entropy of the cipher image using Lian et al.**'**s scheme [24] is 7.978. So the algorithm proposed has a good property of information entropy. In addition, this measure further proves the security of the algorithm.

## 5. Conclusions

This paper presents a hyperchaos digital image encryption technique that is based on bit permutation and dynamic DNA encoding. By using bit permutation, chaos mapping, and the dynamic DNA encoding technique, the scrambling transformation of the pixel locations and the diffusion of pixel values are achieved. The security analysis shows that the algorithm can effectively resist plaintext attacks, differential attacks, and statistical attacks because the algorithm is based on bit permutations and dynamic DNA encoding, and the key space is large; thus, the security is high. Comparisons between this proposed scheme and other researches are just to give us an intuitive and quantitative measures, from which we can infer that the performance of the proposed algorithm has reached the expectation.

## Figures and Tables

**Table 1:** 8 encoding rules.

Rule	1	2	3	4	5	6	7	8
00	A	A	C	G	C	G	T	T
01	C	G	A	A	T	T	C	G
10	G	C	T	T	A	A	G	C
11	T	T	G	C	G	C	A	A

**Table 2:** The XOR operation for DNA sequences.

XOR	A	C	G	T
A	A	C	G	T
C	C	A	T	G
G	G	T	A	C
T	T	G	C	A

**Table 3:** The addition operation for DNA sequences.

ADD	A	C	G	T
A	A	C	G	T
C	C	G	T	A
G	G	T	A	C
T	T	A	C	G

**Table 4:** The subtraction operation for DNA sequences.

Sub	A	C	G	T
A	A	T	G	C
C	C	A	T	G
G	G	C	A	T
T	T	G	C	A

**Table 5:** Correlation coefficients of the proposed algorithm compared with that of Ye's algorithm, X. Wang and Q. Wang's algorithm, and Liu et al.'s algorithm.

	Original image	Encryption image (the proposed algorithm)	Encryption image (Ye's algorithm)	Encryption image (X. Wang and Q. Wang's algorithm)	Encryption image (Liu et al.'s algorithm)
Horizontal direction	0.9646	0.0082	0.0163	0.0097	−0.0152
Vertical direction	0.9304	0.0032	−0.0029	0.0136	0.0140
Diagonal direction	0.9030	0.0150	0.0309	0.0178	0.0218
